# Two-year Clinical and Radiographic Results with a Multidimensional, Expandable Interbody Implant in Minimally Invasive Lumbar Spine Surgery

**DOI:** 10.7759/cureus.7070

**Published:** 2020-02-21

**Authors:** Donald W Kucharzyk, Larry E Miller

**Affiliations:** 1 Orthopedic Pediatric and Spine, DK Orthopedics, Crown Point, USA; 2 Clinical Research, Miller Scientific, Johnson City, USA

**Keywords:** cage, degenerative disc disease, expandable, interbody fusion, luna, lumbar, minimally invasive

## Abstract

Introduction

Minimally invasive spine surgery has become more prevalent in recent years, but the delivery of interbody devices with small footprints may insufficiently restore the disc space, which may lead to instability and non-union. Vertically expandable interbody implants have partially addressed this limitation, but lateral fusion support remains a concern. The purpose of this study was to evaluate two-year safety and effectiveness outcomes with a multidimensional, expandable interbody fusion device (Luna 3D Interbody Fusion System, Benvenue Medical, Inc., Santa Clara, CA) that is delivered through a minimally invasive approach (6-8 mm) that expands in situ to approximate an anterior lumbar interbody fusion footprint of 25 mm diameter.

Material and methods

This was a retrospective, single-center study that evaluated the clinical utility of a multi-expandable interbody cage in patients undergoing posterior or transforaminal lumbar interbody fusion. Key patient-reported outcomes included back pain severity, leg pain severity, and the Oswestry Disability Index (ODI). Radiographic assessments included disc height (anterior, posterior, and average), foraminal height, segmental lordosis, subsidence, implant migration, and pseudarthrosis. Patients were followed at regular intervals over two years postprocedure.

Results

A total of 50 consecutive patients were treated with transforaminal lumbar interbody fusion (TLIF) using the multidimensional expandable implant. Procedural blood loss was minimal (median 200 ml) and the mean hospital stay was 2.1 days. Perioperative complications were reported in three patients and included a dural tear, postoperative ileus, and end-plate violation. All complications were successfully managed conservatively. There were no nerve root injuries or perioperative infections. Over the two-year follow-up period, one case of subsidence and one case of implant migration were noted on radiographic imaging but required no treatment. Comparing the values reported at baseline and two years, the mean ODI score decreased by 61%, back pain severity decreased by 67%, and leg pain severity decreased by 80% (all p<0.001). Comparing radiographic measures from baseline to two years, anterior disc height increased from 7.6 mm to 15.5 mm, posterior disc height increased from 2.9 mm to 10.1 mm, average disc height increased from 5.6 mm to 13.3 mm, foraminal height increased from 12.2 mm to 20.2 mm, and segmental lordosis increased from 6.2 degrees to 14.0 degrees (all changes p<0.001). One case of non-union was observed and the corresponding two-year fusion rate was 98%.

Conclusions

The utilization of a minimally invasive, multidimensional, expandable interbody implant was safe and effective over two years of clinical follow-up. The implant allows the surgeon to re-establish sagittal balance and to provide a larger surface area for fusion as compared to traditional minimally invasive interbody devices.

## Introduction

Transforaminal lumbar interbody fusion (TLIF) is an effective surgical procedure for degenerative disc disease (DDD), spondylolisthesis, and related spinal disorders requiring surgical intervention. The success of a TLIF procedure is reliant on the characteristics of the interbody spacer to restore sagittal alignment and adequately stabilize the painful motion segment. Patients with the greatest improvements in sagittal balance after lumbar fusion also report better clinical outcomes [1-2]. With the recent widespread adoption of minimally invasive TLIF, the risks of iatrogenic injury of the neural structures are decreased relative to open TLIF. In fact, a recent meta-analysis of randomized trials by Miller et al. concluded that the risk of complications, the risk of pseudarthrosis, and the magnitude of pain severity were comparable with minimally invasive and open TLIF, but that minimally invasive TLIF resulted in less perioperative blood loss, shorter hospitalization, and improved functional outcome [3]. Still, due to the limited visualization and narrow access corridor, there are still several shortcomings of minimally invasive TLIF. These include injuries associated with nerve root retraction and complications related to the impacted insertion of an interbody cage, especially in patients with spondylolisthesis, lumbar lordosis, and disc collapse [4-5]. Additionally, the delivery of an interbody implant through a narrow corridor limits its ability to expand the disc space and to provide sufficient surface area to provide adequate columnar support and encourage bony fusion.

Vertically expanding TLIF implants were recently introduced to partially overcome these limitations. Vertically expanding cages are inserted into the disc space in a collapsed configuration and then their height is expanded in situ to restore disc height. While these designs solve issues around cephalad-caudad constraints, the surface area occupied by such implants is no different than with static interbody devices. This, in turn, may negatively impact the ability to place sufficient bone graft into the surgical space and increase the potential risks of subsidence, biomechanical instability, and surgical failure [6-8]. There remains a clinical need for a large footprint interbody device for TLIF.

A new type of interbody implant has been developed that is delivered through a minimally invasive approach and expands multidimensionally in situ, which provides for a larger surface area of approximately 25 mm in diameter, which is comparable to that of interbody cages used in anterior lumbar interbody fusion (ALIF). The purpose of this study was to report two-year clinical outcomes among patients treated in routine clinical practice with a multi-expandable lumbar interbody fusion cage designed to be used in posterior approach fusion surgery.

## Materials and methods

In an extension of a previously published multicenter study of 32 patients treated with the multidimensional, expandable interbody implant who were followed for three months, here we present a retrospective, single-center study that evaluated the clinical utility of a multi-expandable interbody cage over two years of follow-up in patients undergoing posterior or transforaminal lumbar interbody fusion [9]. All study procedures were conducted in accordance with the ethical standards of the institutional research committee and with the 1964 Helsinki declaration and its later amendments. Written informed consent was obtained from all participants included in the study. The study was approved by the Western Institutional Review Board (Study #1160680) and a waiver of consent was granted.

Consecutive patients were retrospectively assessed for study eligibility at a single hospital in the United States. Eligible patients were adults who presented with symptomatic DDD that was unresponsive to at least six months of conservative treatment and received posterior lumbar fusion with the expandable interbody system. Patients diagnosed with an active infection, significant osteoporosis, malignancy, or significant spondylolisthesis or retrolisthesis were excluded from participation. Preprocedural assessments included patient demographics, medical history, physical examination, neurological examination, back pain severity, and leg pain severity (each measured on a 100 mm visual analog scale), Oswestry Disability Index (ODI), and health-related quality of life on the Medical Outcomes Study 36-Item Short-Form General Health Survey (SF-36).

**Figure 1:**
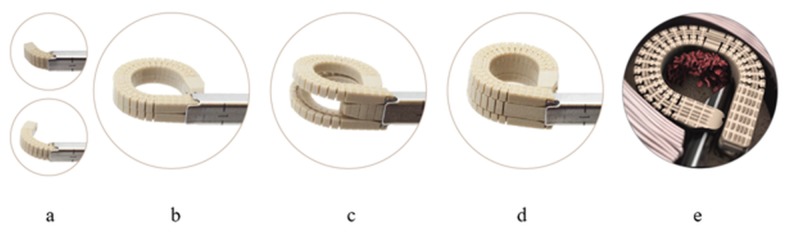
Luna 3D lumbar interbody fusion cage The major steps of deployment include (from left to right): a) initial deployment of the top and bottom components in a curved trajectory from a cannula (top and bottom images), b) completion of top and bottom component deployment in circular configuration, c) initial advancement of middle component between top and bottom components, d) completion of Luna 3D cage deployment, and e) bone graft injection into implant concavity. Adapted from Coe et al. [9]. Luna 3D Interbody Fusion System (Benvenue Medical Inc., Santa Clara, CA)

A detailed description of procedural steps with the expandable device has been published elsewhere [9]. Briefly, patients were positioned prone and secured on the operating table with pads. A 6-8 mm incision was made over the operative level and the soft tissues were dissected to the bone in standard fashion. Tube retractors were placed and the facet joints and pars interarticularis were visualized. Percutaneous pedicle screws were placed under fluoroscopic guidance. A facetectomy was then performed using a posterior or transforaminal approach. Next, a discectomy was performed and the endplates were prepared. All patients received lumbar interbody fusion with a multi-expandable interbody cage via a transforaminal or posterior approach. The interbody cage (Luna 3D Interbody Fusion System, Benvenue Medical Inc., Santa Clara, CA) was cleared for marketing by the United States Food and Drug Administration (US FDA) in November 2014. The device consists of three interlocking polyetheretherketone components, a lock wire and screw, and a graft window where bone graft material is inserted into the cage after in situ device expansion (Figure 1).

**Figure 2:**
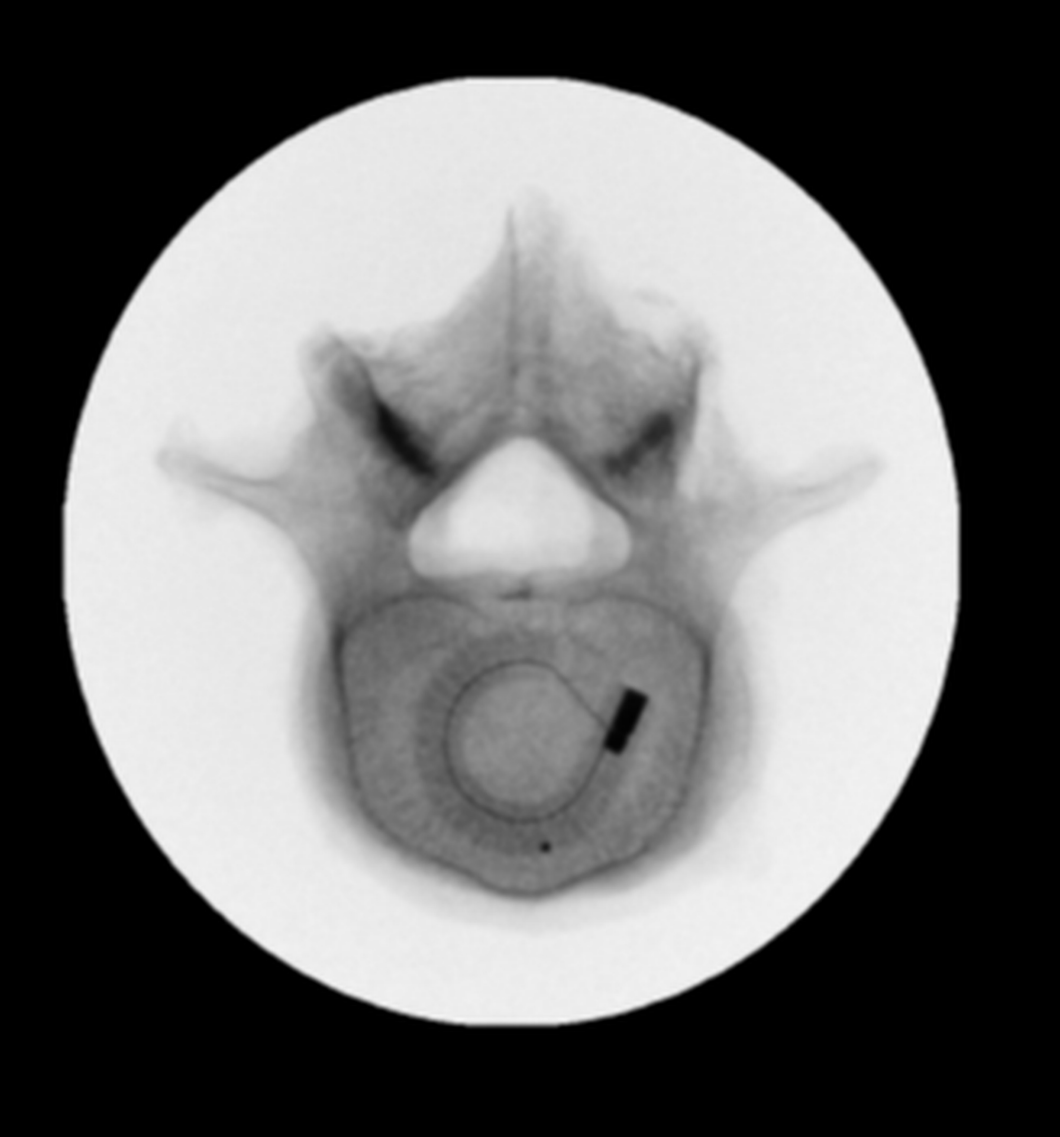
Axial view of lumbar interbody fusion cage in situ demonstrating a footprint of 26x30 mm, which approximates the area of anterior interbody fusion devices. Adapted from Coe et al. [9]

A specialized implant inserter was used to deploy the top and bottom components of the implant into the disc space and the middle component was then advanced between the top and bottom components, increasing the height of the cage. A cage height of 8 mm up to 15 mm (0 degrees and 8 degrees lordosis) may be selected by the surgeon, depending on the patient’s anatomy. The method used to insert the cage allows minimal-impaction delivery, which protects and preserves the vertebral endplates. Upon completion of the insertion, the concavity of the interbody cage was filled with bone graft material according to surgeon preference and specific pathological need. Once grafting was completed, screws and rods were passed bilaterally and set screws were secured. Proper pedicle screw and cage placement were confirmed using fluoroscopy. Surgical closure was then performed in standard fashion. Patients were typically discharged within 24 hours of procedure completion. One of the main advantages of a multi-expandable cage is that an ALIF-like cage sized footprint (Figure 2) can be delivered through a minimally invasive, narrow posterior surgical corridor.

After the procedure, mobilization is undertaken over the first several weeks. Postoperative follow-up visits were scheduled at two weeks, six weeks, three months, six months, one year, 18 months, and two years. At each visit, patient-reported outcomes, neurological status, clinical signs/symptoms, and complications were recorded. Patient-reported outcomes were back pain severity on a 0-100 scale, leg pain severity on a 0-100 scale, ODI on a 0-100 scale, and the Physical Component Summary (PCS) subscore from the SF-36 questionnaire [10-12]. The minimum clinically important difference (MCID) relative to baseline for each patient-reported outcome is at least a 20 mm decrease for leg pain severity, a 20 mm decrease for back pain severity, a 15-point decrease for ODI, and a 5.7-point increase for PCS [13-15]. Four-view X-rays were performed at each follow-up visit and magnetic resonance imaging was performed in patients with pain recurrence, functional decline, or neurological decline. Radiographic assessments included disc height (anterior, posterior, and average), foraminal height, segmental lordosis, subsidence, implant migration, and pseudarthrosis. Fusion was determined using the Brantigan and Steffee criteria via plain X-rays and computed tomography scan if nonunion was suspected [16]. Only solid radiographic fusion counted as a fusion; all other categories were considered pseudarthrosis.

In a power analysis assuming a mean baseline leg pain severity of 80 with a standard deviation of 20, a sample size of 50 patients provided 80% statistical power to detect a decrease of at least 8.0 points from baseline using a two-sided paired t-test with an alpha of 0.05. Patient characteristics were reported using the mean and range for normally distributed continuous outcomes, median and range for non-normally distributed continuous data, and count and frequency for categorical data. The mixed-model analysis of variance was used to analyze longitudinal changes in patient-reported outcomes. Data were analyzed using SAS version 9.4 (SAS Institute, Cary, NC). All statistical tests were two-sided, and statistical significance was set at p<0.05.

## Results

Between November 2015 and October 2016, 50 consecutive patients were treated with TLIF using the multidimensional, expandable implant. The mean patient age was 59 years (range 30-81 years), mean body mass index was 31 kg/m^2^, and 54% were female. Patients commonly presented with multiple lumbar diagnoses; the most common indications for TLIF were radiculopathy (100%), spinal instability (100%), lumbar spinal stenosis (85%), spondylolisthesis (75%), facet arthropathy (75%), and DDD (75%) (Table 1).

**Table 1:** Baseline patient characteristics *Values are mean (range) or count (percent)

Characteristic	Value*
Age, years	59 (30-81)
Female sex	27 (54%)
Body mass index, kg/m^2^	31 (24-26)
Surgical indication	
Radiculopathy	50 (100%)
Spinal instability	50 (100%)
Lumbar spinal stenosis	42 (84%)
Spondylolisthesis	38 (76%)
Facet arthropathy	38 (76%)
Degenerative disc disease	38 (76%)
Lumbar disc herniation	35 (70%)
Retrolisthesis	30 (60%)
Post-laminectomy syndrome	20 (40%)
Number of fusion levels	
One	34 (68%)
Two	12 (24%)
Three	3 (6%)
Four	1 (2%)

Using a minimally invasive, mini-open, or open surgical approach, as appropriate, TLIF was performed at 71 lumbar levels between L2/L3 and L5/S1 in 50 patients. The majority (92%) of fusions were performed at L4/L5 or L5/S1. Single-level fusion was performed in 34 patients, two-level fusion in 12 patients, three-level fusion in three patients, and four-level fusion in one patient. Procedural blood loss was minimal (median 200 ml; range: 75-600 ml) and mean hospital stay was 2.1 days (range 1.0-4.0 days). Perioperative complications were reported in three patients and included a dural tear, postoperative ileus, and end-plate violation. All complications were successfully managed conservatively. There were no nerve root injuries or perioperative infections.

**Figure 3:**
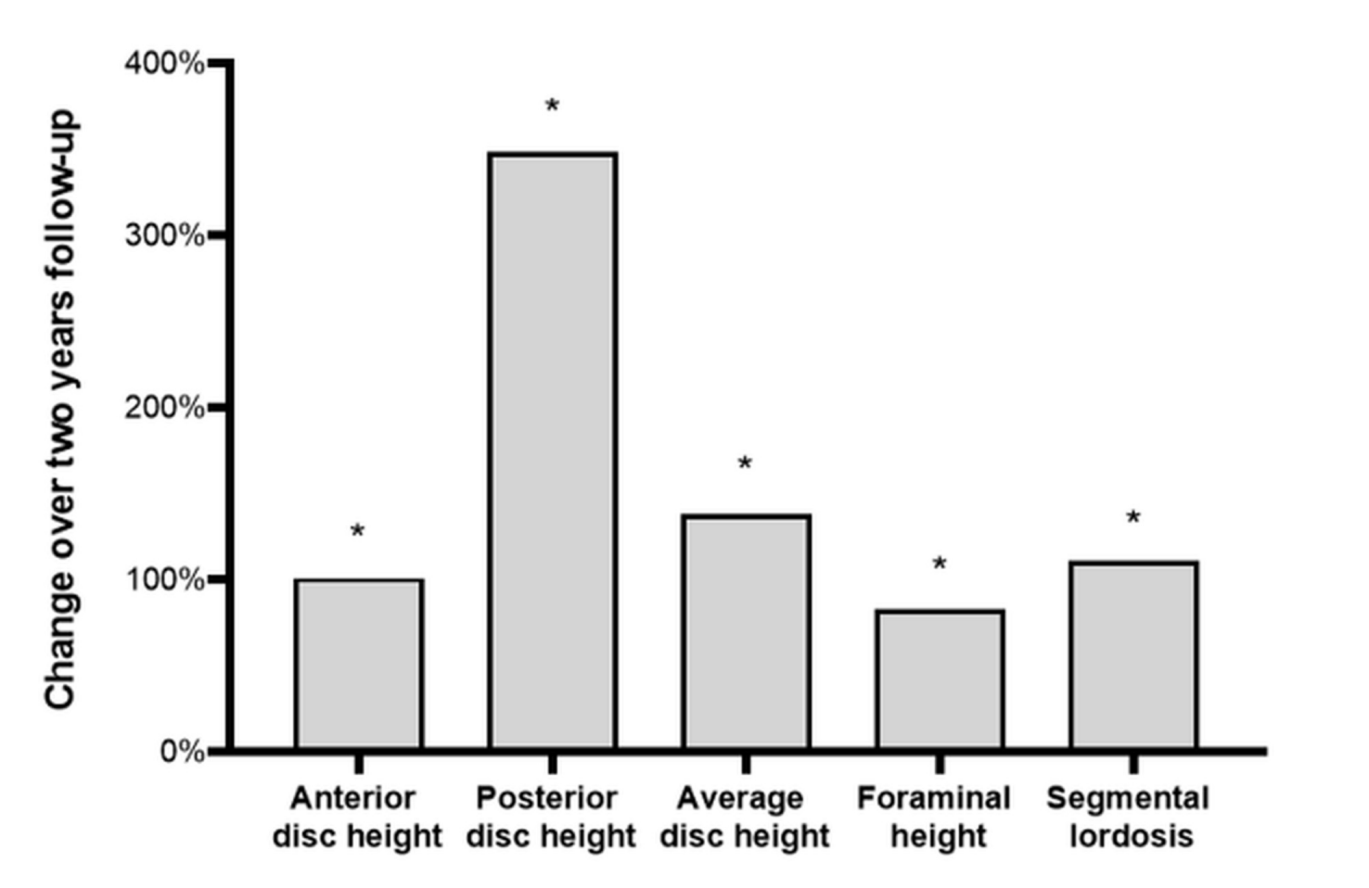
Change in radiographic measures over two years following interbody lumbar fusion with an expandable interbody cage *p<0.001 vs. baseline

**Figure 4:**
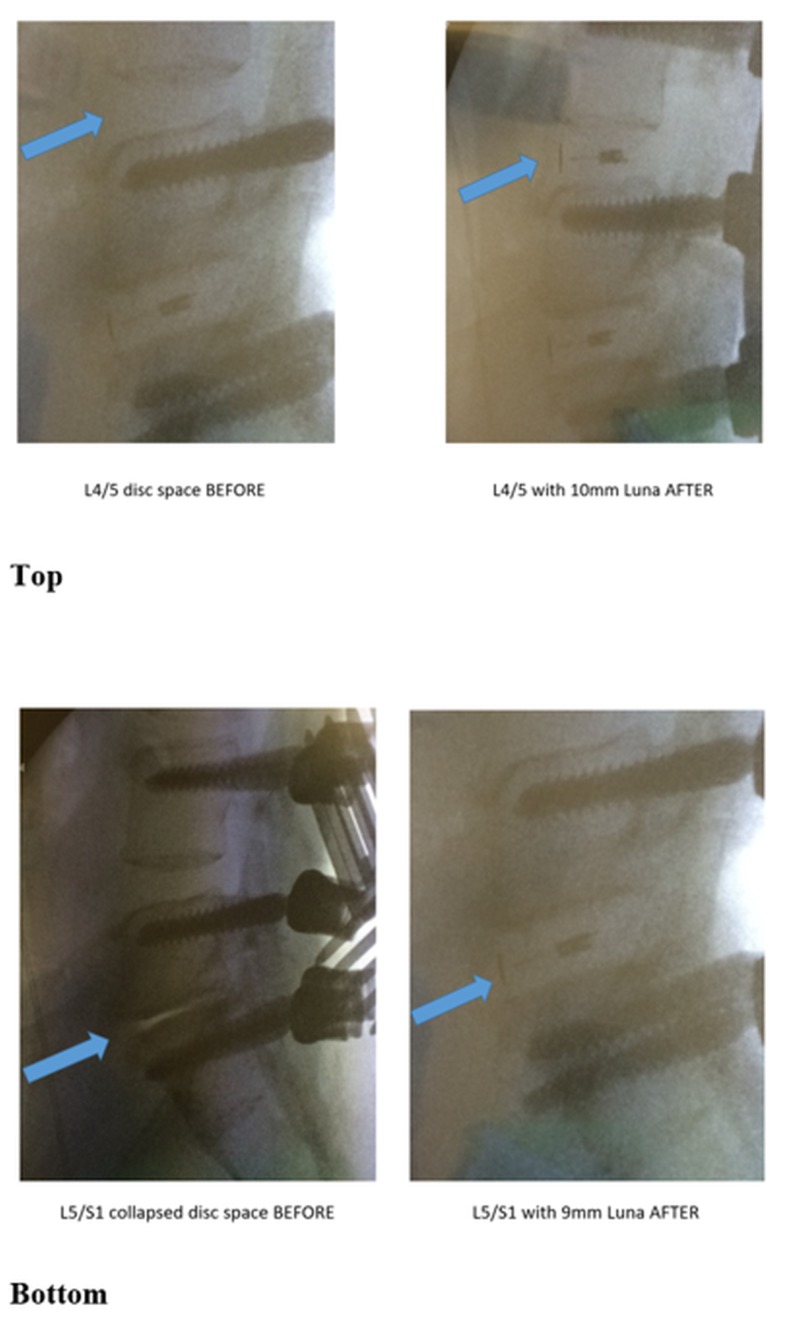
Two-level case demonstrating typical disc height restoration before and after interbody lumbar fusion with an expandable interbody cage at L4/L5 (top) and L5/S1 (bottom)

Over the two-year follow-up period, one case of subsidence and one case of implant migration were noted on radiographic imaging but required no treatment. One case of non-union was observed and was treated with anterior lumbar interbody fusion. The corresponding two-year fusion rate was 98% and this was the only surgical reintervention performed in the series. Comparing radiographic measures from baseline to two years, anterior disc height increased from 7.6 mm to 15.5 mm, posterior disc height increased from 2.9 mm to 10.1 mm, average disc height increased from 5.6 mm to 13.3 mm, foraminal height increased from 12.2 mm to 20.2 mm, and segmental lordosis increased from 6.2 degrees to 14.0 degrees (all changes p<0.001) (Figure 3). Typical increases in disc height following TLIF with the multidimensional expandable implant are demonstrated in Figure 4.

Statistically significant improvements were observed among all patient-reported outcomes through two years. Comparing the values reported at baseline and two years, the mean ODI score decreased by 61% (from 59 to 23). The 36-point mean decrease represented a statistically significant improvement (p<0.001) from baseline and exceeded the MCID of 15 points. Back pain severity decreased by 67% (from 78 to 26) and leg pain severity decreased by 80% (from 81 to 16). Both of these changes were statistically significant at p<0.001 and greatly exceeded the MCID of 20 points. Mean scores on the PCS increased from 27.5 to 38.0, and this 10.5-point increase was statistically significant at p<0.001 and exceeded the MCID of 5.7 points. Ultimately, 93% of patients reported clinical improvement in back and leg pain, 97% reported improvement in leg pain, and 97% had improvement in neurologic function.

## Discussion

Interbody implant techniques are the preferred lumbar fusion construct because of the implant’s ability to provide structural stability and increased fusion rates [17]. The increasing adoption of minimally invasive techniques for spine surgery in recent years has led to significant advancements in instrumentation for lumbar interbody fusion. The capacity to deliver a multi-expandable interbody cage with a large footprint through a narrow surgical cannula represents a significant advancement in spinal surgery technology and fills an unmet clinical need.

The first published clinical experience by Coe et al., reported with the multidimensional, expandable interbody implant, followed 32 patients over three months of follow-up [9]. In that series, there were no neurologic complications and no evidence of subsidence or implant migration. The MCID for back and leg pain was achieved in over 90% of patients and radiographs demonstrated restoration of disc height and restoration or preservation of lordosis over three months of follow-up. Jansen et al. reported one-year clinical results with 30 patients treated with this interbody device [18]. None of the patients showed signs of implant loosening and the total number of adverse events was low (3%). Back pain improved significantly from 81 mm at baseline to 28 mm at one year. The ODI also improved significantly from 58 at baseline to 20 at one year. To the authors’ knowledge, the current study is the first to report patient outcomes over mid-term follow-up sufficient to determine the rates of non-union. From this study, we report that among patients undergoing posterior lumbar fusion in a real-world clinical setting, the use of a multidimensional, expandable interbody cage was safe and resulted in durable improvements in radiographic parameters and clinical outcomes over two years of follow-up.

The ability to deliver an ALIF cage-sized implant using a minimally invasive posterior approach has obvious advantages, such as eliminating the need for an ALIF access surgeon and avoiding iatrogenic injury, since minimal to no nerve retraction is required. The interbody cage engages broader, peripheral sections of the vertebral endplates. In contrast, posterior interbody cages tend to engage smaller, central portions of the endplate. This is advantageous since the risk of subsidence and biomechanical instability is higher when a small-footprint cage is placed in the central or posterior endplate [6-8]. A multi-expandable cage also allows a larger footprint to be obtained than that obtained with a conventional posterior cage, without the need for making a larger incision or having to perform greater nerve root retraction. Finally, the geometry of this multi-expandable cage allows for a large volume of contiguous bone graft to be placed post-expansion, which optimizes the local environment to encourage vertebral body fusion. The ALIF-like performance of the expandable cage is supported not only by the results of the current study but also by biomechanical studies demonstrating that the expandable TLIF cage and ALIF cages provide a similar surface area for bone graft material, increased stiffness imparted by the anterior column, and the ability to restore sagittal alignment [19].

The main strengths of this study included results from real-world clinical practice, patient follow-up over two years postprocedure, and the first known reporting of midterm results with a multidimensional, expandable interbody implant. There were several limitations of this study that warrant further discussion. First, study data were derived from a single center with extensive experience with the interbody implant and it is, therefore, possible that results may differ when performed by less experienced surgeons. Second, there was no control group in this study, which makes a comparison of these results to other studies of interbody cages difficult. Future controlled studies should be conducted to provide comparative evidence in relation to other interbody techniques. Third, the retrospective nature of this study introduces a selection and information bias that may influence study interpretation. Finally, follow-up imaging consisted of radiographs, magnetic resonance imaging, and/or computed tomography, which may introduce variability in the radiographic outcomes reported in the study.

## Conclusions

The utilization of a minimally invasive, multidimensional, expandable interbody implant was safe and effective over two years of clinical follow-up. All measured clinical outcomes greatly exceeded established MCIDs and all radiographic parameters were restored to normal values. The implant represents an improvement over traditional static TLIF cages owing to the ability to deliver the device through a narrow corridor after which the device is deployed in situ to provide an ALIF-like footprint.
